# Challenges and Opportunities in the Management of Onychomycosis

**DOI:** 10.3390/jof4030087

**Published:** 2018-07-24

**Authors:** Julia K. Christenson, Gregory M. Peterson, Mark Naunton, Mary Bushell, Sam Kosari, Kavya E. Baby, Jackson Thomas

**Affiliations:** 1 Faculty of Health, University of Canberra, Bruce, Canberra, ACT 2601, Australia; Julia.Christenson@canberra.edu.au (J.K.C.); Mark.Naunton@canberra.edu.au (M.N.); Mary.Bushell@canberra.edu.au (M.B.); Sam.Koari@Canberra.edu.au (S.K.); 2 Faculty of Health, University of Tasmania, Hobart, TAS 7005, Australia; G.Peterson@utas.edu.au; 3 The Canberra Hospital, Yamba Drive, Garran, ACT 2605, Australia; Kavya.Baby@act.gov.au

**Keywords:** onychomycosis, nail, fungi, infection, treatment, alternative treatment, combination therapy

## Abstract

Onychomycosis is an increasingly common fungal nail infection, chiefly caused by dermatophyte fungi. The disease is notoriously difficult to treat due to the deep-seated nature of fungi within the nail plate, prolonged treatment requirements, poor patient adherence and frequent recurrences. Given the poor efficacy of currently available topical and systemic therapies, there is a renewed interest in exploring alternative treatment modalities for onychomycosis. Natural therapies, physical treatments and various combination therapies have all shown potential for the management of onychomycosis, though research on many of these methods is still in preliminary stages. Further large, well-designed, randomised controlled trials are necessary to confirm the efficacy of these novel treatments in order to make formal recommendations regarding their use in the management of onychomycosis.

## 1. Onychomycosis and Associated Risks

Onychomycosis is a common fungal nail infection that has been estimated to account for about half of all nail diseases [1]. Although not life threatening, fungal nail infections are an important public health concern due to their high prevalence, poor response to therapy and significant clinical, social and financial impact. Risk factors for the development of onychomycosis include advancing age [2], the presence of nail trauma or psoriasis, wearing occlusive footwear, participating in sporting activities like running or swimming, and a history of fungal infection elsewhere on the body [3,4,5]. A rise in the prevalence of onychomycosis over the past few decades has been attributed to a number of causes including an increase in the number of immunocompromised individuals (including people with diabetes) [6], longer life expectancies, increased urbanisation, and use of occlusive modern footwear [7].

Fungal infections are contagious. They can be transmitted to others via direct skin-to-skin contact, but are more often spread through shedding of infected dead skin/nail cells and fomites contaminated with fungal propagules [8]. The secondary spread of fungal organisms within the same individual can lead to the infection of other nails, web spaces, toes and potentially the whole foot. If left untreated, the infection can act as a reservoir for other skin conditions, including atopic dermatitis, urticaria and erythema nodosum, as well as fungal infections in other parts of the body. Onychomycosis can also exacerbate foot problems resulting from other illnesses like diabetes, and in extreme cases can ultimately necessitate lower extremity amputation [9].

## 2. Current Treatments

**Figure 1:**
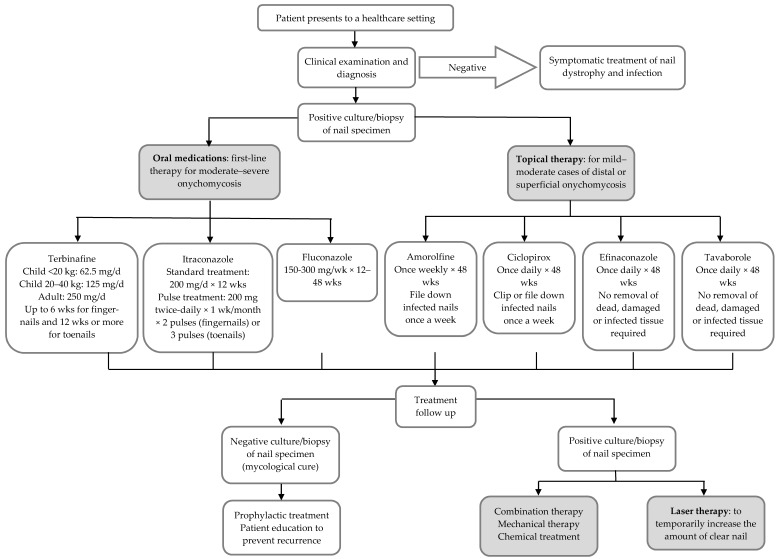
Onychomycosis treatment algorithm (modified from Thomas et al. [4]).

While the range of emerging treatments against onychomycosis is rapidly expanding, most current treatments still fall under either topical agents applied directly to the nails or systemic agents taken orally (Figure 1). Treatment selection depends on the extent and severity of nail changes, the organism responsible, concerns about drug interactions or adverse effects, and success or failure of previous treatments [10]. The severity of infection is assessed based on the extent of nail involvement, degree of nail discolouration and nail plate thickening, onycholysis (separation of nail from nail bed) and pain.

Topical treatments (like amorolfine and ciclopirox) are used for managing minor infections of the nail plate. While topical antifungals have the advantage of causing fewer and less serious side effects, treatment periods are long, and efficacy is limited due to poor nail plate penetration. Clinical trials have shown newer topical agents like efinaconazole 10% and tavaborole 5% to be significantly superior to placebo [11,12]. However, further comparative trials are required to determine their relative clinical efficacy and provide more effective recommendations for patients.

Oral antifungals (like terbinafine, itraconazole and fluconazole) are required for most cases of onychomycosis because of their greater ability to penetrate the nail bed and nail plate. Common side effects of these medications include headaches, gastrointestinal symptoms, nausea and rash [13]. Furthermore, health professionals must be cautious of dangerous drug–drug interactions and increased risk of hepatotoxicity [14]. Terbinafine is currently the preferred oral treatment against onychomycosis, with recent reviews and meta-analyses showing it to be more effective than other treatments [13,15].

## 3. Treatment Challenges

Onychomycosis is notoriously difficult to treat. Achieving complete cure can take as long as 18 months [16] and cure is not achieved at all in 20–25% of treated patients [17]. Furthermore, the disease is associated with very high recurrence rates due to the presence of residual fungal spores or hyphae, with relapse occurring in 6.5–53% of patients [18]. The efficacy of current treatments is limited by the slow growth of toenails, nail keratin thickness preventing penetration of topical and systemic drugs, and survival of fungi in surrounding environments (such as footwear) for long periods. Because of their lack of intrinsic immune function and impenetrable nature, nails are a particularly challenging tissue to cure [19]. Individuals with onychomycosis can experience very long-lasting disease, especially in the absence of effective treatment. An average disease duration of almost 18 years was recorded among 2761 onychomycosis patients in Poland [20].

Recent microbiological advances suggest that fungi, like bacteria, are able to form biofilms, complex sessile microbial communities permanently attached to epithelial surfaces in an extracellular matrix. The presence of a protective barrier created by fungal biofilms may partly explain the high rates of treatment failure, recurrence and relapse seen in onychomycosis [16]. For this reason, it has been suggested that antifungal agents should be tested for effectiveness against fungal biofilms and not exclusively planktonic cells [16].

Susceptibility to onychomycosis increases with the presence of other underlying comorbidities, including chronic renal failure (with dialysis) and renal transplant, immunodeficiency, diabetes, cancer and peripheral arterial disease [3,6]. These comorbidities and associated polypharmacy make some patients ineligible for oral antifungals. Furthermore, systemic side effects associated with oral antifungals can lead to patient nonadherence and treatment failure. To address these issues, new treatment methods are being investigated for onychomycosis that can avoid the pitfalls of standard systemic and topical therapies. Natural therapies, physical treatments like photodynamic therapy [21] and laser therapy [22], and various combination therapies are among treatment avenues currently being explored by researchers and clinicians to improve cure rates in onychomycosis.

## 4. Natural Therapies

Given the challenges associated with available topical and systemic agents, there is a renewed interest in exploring alternative natural treatments for onychomycosis [19]. Natural onychomycosis therapies may have some important advantages over current treatments. They have the potential to be less expensive, particularly if classified as over-the-counter or complementary medications. Furthermore, they have shown low levels of adverse reactions in human trials (mostly skin irritation or mild pain) (Table 1), suggesting that these medications could be safer and more tolerable than standard therapies. Because of the complex nature and composition of bioactive constituents present in plant-based treatments, natural treatment modalities may carry a lower risk for the development of fungal resistance [23]. These qualities indicate potential for natural therapies to be employed as prophylactic agents against onychomycosis.

While a number of natural remedies have shown promising antifungal activity in vitro, clinical trials on these treatments are as yet in early stages. For example, essential oils like tea tree oil have demonstrated antifungal activity in vitro [24,25], but their efficacy has not been adequately confirmed against onychomycosis in human trials [26] (Table 1). Most human trials investigating natural therapies for onychomycosis have been small-scale pilot studies that are not directly comparable with trials on standard treatments due to differences in design and methodology.

A six-month clinical trial investigated *Ageratina pichinchensis* (AP), a plant used in traditional Mexican medicine, as a topical treatment for onychomycosis [27]. Results showed AP 10% lacquer to be equivalent to ciclopirox 8% lacquer in terms of effectiveness against onychomycosis [27], and a follow-up trial showed that higher concentrations of AP can improve patient outcomes [28]. Natural Coniferous Resin (NCR) lacquer showed a mycological cure rate of 65% in a preliminary observational study [29], but only 13% in a randomised controlled trial. The clinical trial showed nine months of daily-applied NCR lacquer to be about as effective as weekly-applied amorolfine 5% lacquer, but less effective than three months of once-daily oral terbinafine (250 mg) [30].

Recent pilot studies suggest potential for other natural compounds as therapeutic options for onychomycosis. For example, propolis, an adhesive resinous compound produced by honeybees to seal and protect their hives from pathogenic agents, exhibits significant antifungal and anti-biofilm activities in vitro [31]. A pilot study assessing treatment of onychomycosis with topical ethanol propolis extract for six months showed complete mycological and clinical cure in 56.3% (*n* = 16), with no adverse events reported [31]. The over-the-counter topical cough suppressant Vicks VapoRub^®^ also showed some effectiveness against onychomycosis in a small-scale uncontrolled pilot study (*n* = 18; mycological cure rate 27.8%; partial/complete clinical cure rate 55.6%/27.8%) [32]. This product contains a combination of active and inactive ingredients, including camphor, eucalyptus oil, menthol and thymol, that have shown in-vitro activity against dermatophytes. It has been suggested that Vicks VapoRub^®^ may be a suitable onychomycosis treatment for people living with HIV due to its low cost, minimal side effects and compatibility with antiretroviral medications [33].

Further large well-designed randomised controlled trials are necessary to determine the efficacy of natural treatments and make formal recommendations as to their use in onychomycosis.

## 5. Physical Treatments

Photodynamic therapy (PDT), plasma therapy and laser treatments are physical therapies that have recently gained traction in the treatment of onychomycosis following the success of in-vitro studies [10]. In PDT, light within a defined narrow spectrum is used to excite a photosensitising agent applied directly to the target area and absorbed by the target organism, resulting in the formation of reactive oxygen species that selectively destroy infected tissue [38]. Photosensitising agents currently being investigated for onychomycosis treatment include the protoporphyrins aminolevulinic acid and methyl-aminolevulinate, the haematoporphyrin derivative Photogem^®^, and rose bengal and methylene blue dyes [21,39,40]. Studies investigating PDT to treat onychomycosis in vivo are still largely limited to case reports, though a few small clinical trials have been performed [40].

A 2016 systematic review of five in-vitro and 12 in-vivo studies (mostly case reports and unrandomised human trials) suggested that PDT may be able to provide a therapeutic benefit for onychomycosis, with minimal (and no systemic) side effects [21]. Reported side effects (mild pain, burning, erythema, oedema and blistering) were well tolerated and resolved within a few days. A 2014 randomised clinical trial assessing the efficacy of biweekly PTD showed a clinical cure rate of 90% at six months (and 80% at 12 months) in 40 patients with toenail onychomycosis [41]. Affected nails were treated for six months with 2% methylene blue and irradiated with noncoherent red light from an LED device (630 nm, 18 J/cm^2^). Those in the comparative group receiving oral fluconazole responded poorly, while the PDT treatment was effective and well tolerated. While outcomes of early trials have been promising, further investigation is needed to confirm the efficacy and safety of PDT in treating onychomycosis.

An important concern with PDT is the requirement for frequent and lengthy clinic visits (up to several hours each), which may compromise patient compliance. Patients may need to attend up to 12 treatment sessions across six months, compared to only one or two doctor’s appointments while undergoing oral or topical therapy [40]. Also, successful treatment appears to require pretreatment of the affected area to improve penetration of the photosensitising agent into the nail plate. Studies to date have employed pretreatment methods including application of urea ointment to soften the nail, microabrasion or even complete removal of the nail plate [21].

Another physical treatment being investigated for onychomycosis is plasma therapy, which involves applying nonthermal plasma to the infected nail surface. Pulses of strong electric field are used to generate the plasma which ionizes surrounding air molecules to produce the antifungals ozone, nitric oxide and hydroxyl radicals [10]. Nonthermal plasma has exhibited antifungal activity in vitro [42,43], and a 2017 pilot study on a plasma device to treat onychomycosis (*n* = 19) showed the treatment to be safe and effective, yielding a clinical cure of 53.8% and mycological cure of 15.4% [44]. Plasma therapy has similar advantages to PDT in that it is noninvasive, compatible with comorbidities and does not interact with other drugs. Larger-scale trials are required to determine whether plasma therapy can successfully treat onychomycosis in practice.

Laser therapy is a nonpharmacologic treatment thought to induce fungicidal activity through a photothermal effect on nail fungi using the principle of selective photothermolysis. The mechanism of action is not yet well understood, but one theory is that laser energy is preferentially absorbed by fungal mycelia in the affected tissue, resulting in a rapid elevation in temperature and fungal cell death [45]. Lasers have received regulatory approvals from the US Food and Drug Administration (FDA) and Australian Therapeutic Goods Administration (TGA) to treat fungal-infected nails [22,46]. However, as emphasised in a recent review by Gupta et al. [22], the FDA approval for laser treatment of onychomycosis restricts its use to aesthetic endpoints (for “the temporary increase of clear nail”) rather than medical treatment goals (such as mycological or complete cure).

Various types of lasers have been investigated for use in the treatment of onychomycosis. Those most extensively studied for this purpose are neodymium-doped yttrium aluminium garnet (Nd:YAG) laser devices [47], which can be modified to operate in continuous, long-pulsed, Q-switched or potassium titanyl phosphate modes. Nd:YAG lasers are typically used to emit a 1064-nm wavelength, though both shorter and longer wavelengths are available. Onychomycosis has also been treated with fractional carbon dioxide lasers, diode lasers and erbium:glass lasers [47]. Cost is a major factor when considering laser and light treatments as they are not typically covered by health insurance plans. As such, treatments can cost from US$400 to US$1200 per session [48], compared to only $10 for a 12-week course of terbinafine (or $53 if the cost of liver function tests is included) [49].

Recent reviews on laser treatment of onychomycosis have concluded that the evidence for its effectiveness is limited and of poor methodological quality [22,50,51]. Current findings are largely based on studies with small sample sizes, differing definitions for outcome measures and a lack of control groups. A major point of difference between studies is that some evaluate endpoints using patients as the unit of analysis with one target toenail, while others use nails as the unit of analysis and include multiple nails per person, potentially resulting in overestimation of treatment efficacy [22]. In their 2017 review of 21 studies, Gupta and Versteeg [22] found that laser therapy resulted in clinical improvement in 36% of patients (based on five studies) and 67% of nails (based on nine studies), but clinical cure (100% clear nail) in only 13% of patients/nails (based on two/six studies). Mycological cure, a standard endpoint of treatment, occurred on average in 11% of patients (two studies) and 63% of nails (three studies). Therefore, laser therapies may be somewhat effective in achieving cosmetic endpoints in onychomycosis, but they do not equal or exceed the efficacy of current topical and oral treatments in terms of achieving medical endpoints [22]. Based on the present evidence, lasers should not be considered an effective first-line therapy for onychomycosis.

## 6. Combination Therapy

Combining onychomycosis treatment options can increase their efficacy and improve patient outcomes, possibly due to synergy, differing mechanisms of action and restricting the effect of fungal resistance [52]. In their 2017 review, Gupta and colleagues [52] summarised studies combining oral and/or topical drugs for onychomycosis, revealing that combination therapy is sometimes (though not always) more effective than monotherapy. Most studies combined oral terbinafine or itraconazole with topical amorolfine 5%, ciclopirox 8% or terbinafine.

Many combination therapies for onychomycosis are based on improving drug delivery to the infected nail via adjunct physical, mechanical or chemical treatments, including nail filing, nail trimming, debridement, chemical avulsion and the drilling of small holes into the nail plate (microporation) [52]. A promising emerging adjunct treatment is iontophoresis, which may be combined with topical therapies to improve their effectiveness. The method involves passing a small electrical current through a biological membrane that allows transportation of hydrophobic or charged drug molecules. Early investigations indicate that terbinafine can be delivered at higher concentrations using iontophoresis, improving the ability of the drug to eliminate fungi [53,54].

Recent studies have looked at combining topical therapies and laser treatments to improve drug penetration into the nail. One randomised clinical trial evaluated the clinical efficacy and safety of weekly applications of amorolfine 5% lacquer, either as a monotherapy (*n* = 64) or in combination with four 1064-nm Nd:YAG laser treatments (*n* = 64) over 16 weeks [55]. At 16 weeks, the combined therapy group showed a significantly higher cumulative cure rate than the control group (71.9% vs. 20.3%, *p* < 0.0001). Combined therapy was generally well tolerated (with patients experiencing transient discomfort), showing potential for use as an alternative treatment for patients with contraindications to systemic antifungal agents. Other small-scale pilot studies have shown similarly promising results for twice-weekly amorolfine 5% lacquer combined with six fractional erbium yttrium aluminum garnet (Er:YAG) laser treatments [56], and daily amorolfine cream combined with three fractional carbon-dioxide laser treatments (no control group) [57].

Preliminary investigations of laser therapy plus oral medications to treat onychomycosis have shown that long-pulsed 1064-nm Nd:YAG laser treatments, combined with oral terbinafine for 12 weeks, resulted in significantly higher mycological and clinical clearance rates than either treatment alone [58], while a combination of similar laser treatments with oral itraconazole was statistically superior to the itraconazole alone only when the onychomycosis was classified as ‘severe’ rather than ‘mild or moderate’ [59]. Further controlled studies are required to evaluate whether these combination therapies have sufficient merit to recommend in practice.

The limited safety profile of oral antifungals, poor nail penetration of onychomycosis drugs, and mediocre therapeutic efficacies of conventional treatment modalities signal the need for new treatment options and therapeutic inventions. Emerging treatments include new topical, photodynamic and laser therapies, and combination therapies appear to hold promise for improving patient outcomes. It is likely that in the imminent future, additional topical agents, including those which are currently approved for other superficial fungal infections (such as tinea pedis), will become available to onychomycosis patients. Preliminary trials on natural topical treatments have revealed some promising agents which may be useful as safe alternatives to current therapies for the long-term management of onychomycosis, especially in patient populations where standard oral therapy is limited. However, the current evidence is insufficient to make broad clinical recommendations.

## Figures and Tables

**Table 1:** Clinical trials investigating natural therapies against onychomycosis.

Study	Patients and Treatment	Outcomes	Treatment Comparisons and Adverse Events (AE)
Buck et al. 1994 [34] Randomised, double-blind trial	117 pts with DLSO. Tea tree oil (TTO) 100% (*n* = 64) Clotrimazole (CL) 1% solution (*n* = 53) Applied twice daily for 6 months	Mycologic cure TTO 18% Clinical assessment TTO 60% Patient assessment TTO 56% Mycologic cure CL 11% Clinical assessment CL 61% Patient assessment CL 55%	No statistical differences between treatments Most common AE were erythema and irritation (7.8% TTO)
Syed et al. 1999 [35] Randomised, double-blind, placebo-controlled trial	60 pts with DLSO **Butenafine hydrochloride (BH) 2% and TTO 5% cream** (*n* = 40) **Tea tree oil 5% cream** (*n* = 20) Applied three times daily for 8 weeks, with nails debrided between weeks 4 and 6. Final follow up at 36 weeks	Complete cure BH + TTO 80% Complete cure TTO 0%	BH + TTO was statistically superior (*p* < 0.0001), and mean time to complete healing was 29 weeks No AE in TTO group. Mild skin inflammation in 4/40 pts in active BH + TTO group
Auvinen et al. 2015 [30] Prospective, randomised, controlled, investigator-blinded trial	73 pts with toenail onychomycosisNatural Coniferous Resin (NCR) lacquer (*n* = 23) Applied once daily for 9 months Amorolfine (A) 5% lacquer (*n*= 25) Applied once weekly for 9 months Oral terbinafine (T) 250 mg (*n*= 25) Taken once daily for 3 months	Mycologic cure NCR 13% Partial cure NCR 30% Complete cure NCR 0% Mycologic cure A 8% Partial cure A 28% Complete cure A 0% Mycologic cure T 56% Partial cure T 36% Complete cure T 16%	At 10 months follow up, oral T was significantly superior to NCR and A in terms of mycologic cure and clinical outcomeNo AE in NCR or A groups. 2 pts with diarrhoea and rash in T group
Romero-Cerecero et al. 2008 [27] Randomised, controlled, double-blind trial	110 pts with toenail onychomycosis *Ageratina pichinchensis* (AP) 10% lacquer (*n* = 55) Ciclopirox (CL) 8% lacquer (*n* = 55) Applied once every three days for 4 weeks, twice a week for 4 weeks, then once a week for 16 weeks. Lacquer removed weekly	Clinical effectiveness AP 71.1% Mycologic cure AP 59.1% Treatment compliance 95.9% Clinical effectiveness CL 80.9% Mycologic cure CL 63.8% Treatment compliance 100%	No statistical difference between treatments No severe AE reported
Romero-Cerecero et al. 2009 [28] Randomised double-blind trial	122 pts with DLSO AP 12.6% lacquer (*n* = 62) AP 16.8% lacquer (*n* = 60) Applied once daily for 6 months	Clinical effectiveness/complete cure 12.6% AP 67.2% Clinical effectiveness/complete cure 16.8% AP 79.1% (no clinical manifestation in toenails, considered healthy)	The 16.8% AP lacquer formulation possessed a higher effectiveness than the 12.6% AP lacquer formulation (*p* = 0.01)No AE reported
Menéndez et al. 2011 [36] Randomised, controlled, single-blind trial	400 pts with onychomycosis OLEOZON^®^, ozonized sunflower oil * (OSO) (*n* = 200) Ketoconazole cream (KC) 2% (*n* = 200) Applied twice daily for 3 months, with filing and massage of affected nails upon treatment application	Complete cure OSO 90.5% Improvement OSO 9.5% Complete cure KC 13.5% Improvement KC 27.5%	After 3 months, OSO was more effective compared to KC (*p* < 0.00001) At 1 year follow up, relapse had occurred in 2.8% of cured pts in OSO group and 37.0% of cured pts in KC groupNo AE reported
Parekh et al. 2017 [37] Randomized, placebo-controlled, double-blind, parallel trial	28 pts with severe tinea (*n* = 18) or onychomycosis (*n* = 10) Calmagen^®^ * cream or lotion (C) (*n* = 14) Placebo (P) (*n* = 14) Applied for 12 weeks	Mycologic cure C (13/14) 92.8% Clinical cure C (14/14) 100% Mycologic cure P (0/14) 0% Clinical cure P (0/14) 0%	There was a significant difference in mycologic cure rate between both arms (*p* < 0.0001) No AE reported

DLSO = Distal lateral subungual onychomycosis; pts = patients; * with active ingredient AMYCOT^®^, a bioactive extract derived from *Arthospira maxima* (*Spirulina*); adapted from studies reviewed by Halteh et al. [19].
